# “Time’s up” – staff’s management of mealtimes on inpatient eating disorder units

**DOI:** 10.1186/s40337-015-0052-4

**Published:** 2015-04-01

**Authors:** Trine Wiig Hage, Øyvind Rø, Anne Moen

**Affiliations:** Institute for Health and Society, University of Oslo, Oslo, Norway; Regional Department of Eating Disorders, Division of Mental Health and Addiction, Oslo University Hospital, Oslo, Norway; Institute of Clinical Medicine, University of Oslo, Oslo, Norway

**Keywords:** Eating disorders, Mealtimes, Practice based studies, Script theory, Qualitative methodology

## Abstract

**Background:**

Refeeding and normalizing eating behaviour are main treatment aims for individuals admitted to inpatient eating disorder units. Consequently, mealtime activities are specific, everyday activities, serving a clear therapeutic purpose, despite numerous challenges for both staff and patients. Few studies have specifically addressed staff involvement, interactions, and management activities to structure mealtimes. In this study, we investigated the structure of mealtime activities on inpatient eating disorder units, and identified associated staff behaviour.

**Methods:**

Descriptive and exploratory qualitative study using video observations to investigate the structure of mealtimes and staff management of mealtime activities. Forty main meals were video recorded and the observational data were analysed using interaction analysis.

**Results:**

An initial analysis during data screening identified three main parts of the meal: ‘*pre-eating’*, ‘*eating*’, and ‘*meal completion’*. For each part, a regular pattern of activities occurred which were associated with staff behaviour.

**Conclusions:**

Increased awareness amongst staff regarding how they manage the meal and act through a clear internal structure can help staff members to further explore their behaviours and collaboration during mealtimes, and also contribute to improved interaction with patients during the various phases of the meal.

## Background

The importance of food in the treatment of eating disorders (ED) is well established. In his classic description of Anorexia Nervosa (AN) in 1874, Sir William Gull stated: “I don’t prescribe medicine, because nursing and food are more important than anything else”. Interventions targeting weight gain and normalized eating behaviour are still key treatment goals. Eating behaviour, directly or indirectly related to weight restoration, is a strong predictor of clinical outcome [1-3]. It is also well known that eating is an anxiety-producing activity for individuals admitted to an eating disorder unit (EDU). Perhaps unsurprisingly, weight gain is often associated with negative emotions like fear and anxiety [4,5] and eating is a challenging activity for ED patients. There are several possible explanations for this. Eating, and the consumption of fat foods in particular, may be directly associated with weight gain. Some patients experience eating as contaminating the body, and differentiate between “clean” and “unclean” foods [6,7]. Mealtime is similarly perceived as challenging by staff members, sometimes described as a battleground indicative of an “us” and ”them” culture between staff and patients in the dining room [8,9]. Although widely acknowledged and well established as an important therapeutic activity, there is still a notable lack of empirical research exploring and reporting central aspects of meals and activities during mealtimes on inpatients EDUs [8]. Despite difficulties associated with mealtimes from a staff perspective, and some knowledge about the external structure and organization of mealtimes, there is a scarcity of research investigating staff activities and management during mealtimes on inpatient EDUs.

Inpatient EDUs treat the most critically ill patients suffering from an ED. Patients are usually severely underweight and diagnosed with AN. The staffs’ main responsibilities include food preparation, monitoring the client’s food intake and their activities during the meal, supporting and supervising the patients, as well as providing opportunities to challenge their eating difficulties in a safe and structured environment [10,11]. The organization of treatment, as expressed through collective processes with an emphasis on common structures and guidelines, informs important daily activities. Mealtimes on inpatient EDUs are, in addition to individual and group-based treatment, key therapeutic activities, collective in nature and managed by staff members, typically nursing/milieu staff [10]. There are no standardized guidelines concerning the “how to” of mealtime management on inpatient EDUs [12]. Accumulated experiences and local, informal routines are common. Development of guidelines pertaining to mealtime on EDUs should ideally be informed by empirical studies conducted to assess current practices to inform how to better support the patients in reaching the goal of normalizing eating behaviours and a healthy weight [8]. Still mealtimes typically have an explicit structure. Boundaries for the patients concerning food and eating are seen as conducive for behaviour change, coupled with strategies to refrain from daily discussions and avoid that the topics of food and eating take up too much time and attention [10]. Nursing staff working with ED patients underline the importance of clear eating rules for meals, and direct supervision of eating habits. Also, staff members often assume greater responsibility for the meals in the beginning of a patient’s inpatient treatment period, then gradually granting more autonomy to the patients [13]. One study has assessed mealtime protocols on ED units, revealing large variations in how mealtimes were practiced and organized. They also reported that most units had an external structure to organize mealtimes, e.g. fixed time limits for the duration of meals, specified menu choices and conventions for acceptable mealtime behaviour or etiquette at the shared meal [8].

Mealtimes are recurring events on a daily basis. Such regular events can be viewed as routinized or habituated behaviour, for which organizations and their members establish shared meaning by guaranteeing mutually, fulfilled expectations [14-16]. These types of activities can be studied and elaborated using script theory. Scripts represent knowledge of events and their sequences, and serve to guide expected behaviour for a particular context or well-known situations. Scripts are therefore abstract knowledge structures that describe the sequence of actions in a specific situation. The script is evoked when entering the relevant situation. The majority of scripts have goal attainment as an objective [16]. To assist patients on the EDU with achieving primary treatment goals, behavioural scripts for staff participating in meals should be centred around refeeding and achieving a normalized pattern of eating. Patients with AN often, in addition to inadequate meals, have particular rituals or rules regarding how they eat, e. g., eating specific foods in a specific order [17]. In order to regain a normalized eating pattern, patients must learn what constitutes a normal meal and how it should be eaten. This represents the duality of an everyday activity and an activity serving a clear therapeutic purpose. This duality is likely to evoke different scripts within the overall script for a “meal on EDU”, used by staff members participating in meals: one script is directed at the goal of achieving meal completion and ultimately weight gain, and one script that is directed at the goal of normalized eating behaviour.

To improve our knowledge and inform guidelines, we aimed to determine the structure of a meal, revealing the operating scripts. To do so, we observed mealtime behaviour and staff interactions. The clinical aims of this study were to 1) Describe empirically and explore the internal structure of mealtimes on EDUs, and 2) Describe how these structures, conceptualised as *scripts* affect staff members’ engagement in and management of mealtimes.

## Methods

We designed a descriptive-exploratory, qualitative study to investigate teamwork and interaction on inpatient EDUs.

### Setting

The setting for this study was a specialized, 12-bed inpatient EDU which offers treatment to adolescents and adults aged 16 years and older diagnosed with an ED. Nearly all the patients admitted are diagnosed with AN, with a mean pre-treatment body mass index (kg/m^2^) of 14–15. All admissions are scheduled, and the length of stay varies from 4 months to 14 months. The average length of stay is 6 months. Both voluntarily and sectioned patients are admitted. The treatment programme is set up as a multidisciplinary treatment approach, combining group therapies, individual therapies and dietetic management in line with national guidelines [18]. The programme incorporates a range of different interventions. Dietetic aspects of the programme include mealtime supervision, and individual counselling (if necessary). During the time admitted to the EDU, the patient is required and expected to take part in everyday activities, as part of the therapeutic interventions and treatment. All patients are required to eat four main meals per day: breakfast, lunch, dinner and an evening meal, in the dining room. All patients have their own, tailored individual meal plan based on a “basic meal plan” as determined by the dietician. Weight gain goals are 0,5-1,0 kg per week. Meals are prepared in the EDU’s kitchen, across the hall from the dining room. The dining room is set up with two tables: a “self-serve” and a “be-served” table. At the “self-serve” table, patients help themselves to food, and at the “be-served” table, the patient’s meal is prepared and pre-measured in the kitchen. Seating depends on the patient’s condition, the level of support and attention required, and on how much individual responsibility they can assume for their own food intake. Most patients eat either a full or half basic meal plan, and any individual adjustments reflect on the progress of treatment. If a patient is unable to finish the meal, a fortified nutritional drink supplement is offered. Each meal lasts 30 minutes, followed by a 30-minute mandatory resting period, where patients and staff members spend time together in the living room. There are staff members present at all times during the meal and resting period. Staff members can choose to eat or not when sitting with the patients.

### Data collection

Data collection included video recording of the four main meals: breakfast, lunch, dinner and evening meal. A total of 40 meals were filmed, 10 of each meal type, yielding 20 hours of video observation. Each recording lasted 30 minutes. Two to three meals were observed every weekday during the data collection period. The data was collected between April 2013 and June 2013. The use of video made it possible to record both verbal and nonverbal interaction and communication during the observed situations [19]. To capture all possible variations of meals types, all four main meals were subject to observation. A camera was mounted in the dining room before each meal and was turned on by either the principle investigator or a staff member a few minutes prior to the start of the meal. An external microphone was placed on the dining table, to ensure high quality sound. The dining room is quite large, and therefore, only one table per meal was observed to obtain high quality data for verbal and nonverbal interaction. The patient group varied during the data collection period, and we observed meals where 2 to 7 patients per table attended each meal. In line with study goals to focus primarily on staff behaviour, the table with the most staff members present was selected for video recordings. For most meals, this was the “be-served” table, seating patients who required the most staff support. A total of 3–4 staff members were typically present per meal, divided between the two tables.

### Participants

Staff members were recruited for participation in the study via an information meeting at the unit and all staff members consented to participate. A total of 22 staff members, 19 females and 3 males, consented to participate in the study. Mean age of participants was 39,9 years, ranging from 26 years to 52 years. Their average work experience at the observed EDU was 4,7 years. All had a minimum bachelor-level education. Nine were registered nurses and 13 were social workers, child welfare officers, or similar.

### Ethics

The study was conducted in accordance with the Helsinki convention, following principles of informed consent, anonymity and right to withdraw from the study. Approval from the Institutional Review Board at the hospital was obtained. Prior to the study, separate information meetings were held for the patients admitted to the unit during the data collection period. The patients were explained the focus of the study; namely, how meals are structured and managed by staff members. Only one patient did not wish to participate at mealtimes when staff members were subject to observation, and we set up special arrangements to avoid interference with the therapeutic goals for this patient.

### Data analysis

The empirical data was analysed according to principles of Interaction Analysis (IA) [17]. This approach to analysis is specifically suited for the analysis of video material, and observations of complex work situations involving several participants. The main goal of our analysis is in line with IA: “to identify regularities in the ways in which participants utilize the resources of the complex social and material world of actors and objects within which they operate” ([20]:3).

We focused on a) how participants make the structure visible to themselves and each other, b) temporal organization of ‘moment to moment’ , real-time interaction, and c) repetitive, routinizing aspects of activity and with their variability. The analysis began by reviewing all recorded meals to establish an overall sense of the data material. Nvivo 9 qualitative research software [21] was used for organizing and structuring the data material. The next step comprised choosing sequences for transcription and in-depth analysis. Foci of analyses were sequences describing the internal structure and segmentations of events within each meal, and how the activities were temporally organized within the externally – imposed timetable. All excerpts selected as relevant to illustrate the chosen foci of analysis were transcribed, organized into nodes and further organized into categories. Video excerpts were watched together with the supervisors during the analysis process to discuss the material and verify the results.

## Results

The observed meals had specific *official* beginnings and endings, according to the externally imposed time limit of 30 minutes per main meal. Initial screening of the data material revealed a more complex internal structure. We identified three main structuring events within each meal:

*Pre-eating:* Staff members facilitate and assist patients to take their food and bring it to the table.

*Eating*: Staff members and patients are seated around the table. Patients eat their meals, with staff members supervising and supporting them.

*Completion:* Patients finish their meals, and staff and patients prepare to leave the dining room*.*

The presentation of findings below is organized according to this structure. The detailed examples are data that reflects key aspects and activities of each structuring event.

### Pre-eating

**Figure 1:**
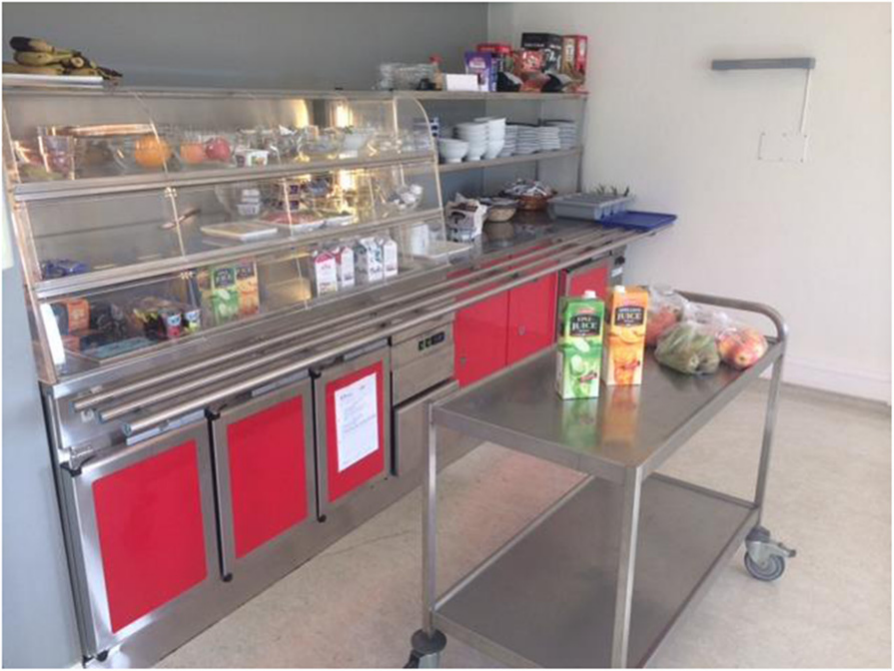
**Picture of serving area.** The serving area consists of a trolley where the food for each meal is placed.

**Figure 2:**
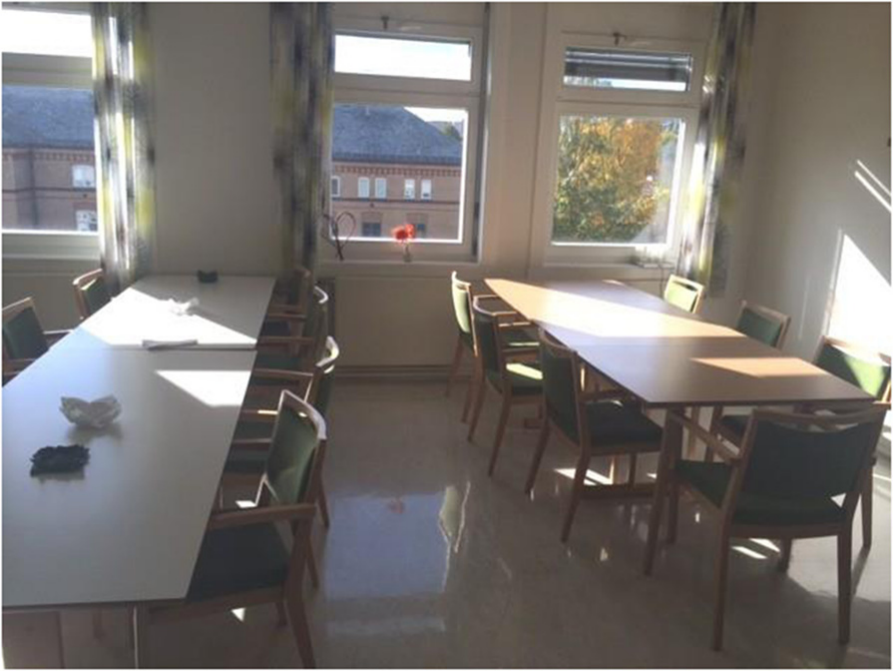
**Picture of dining area.** ‘Be-served’ table to the right. ‘Self serve’ table to the left. The buffet contains an assortment of drinks and sandwich fillings.

This event starts immediately after the official beginning of the meal. ‘Pre-eating’ is the period where staff and patients prepare for the actual eating. Most of the activity in ‘pre-eating’ takes place in the serving area with a serving trolley and a buffet where the food for each meal is placed, close to the dining area of the room (see Figures 1 and 2).

The excerpt below is from a lunch meal. The observed activity occurs at the “be served” table. There are three staff members and four patients present. Patients line up in front of the trolley. Staff member 1 stands next to the trolley, observing the patients either getting their pre-prepared meals or supervising patients helping themselves according to their meal plans. Staff member 3 stands next to the be-served table.

**Excerpt 1, illustrating*pre-eating.***

*SM: staff members. Italics: verbatim transcription of interaction. Text in [brackets]: researcher’s understanding/interpretations. ?: unclear receiver of utterance.

Time span; 0.00-4.59.

1. SM2 is seated at be-served table, on a middle seat. At one point, SM1 walks towards the corner of the room, fetches some napkins from a shelf, then returns to the trolley. Simultaneously, SM3 is moving towards the door, but stops, and turns her body towards the trolley. When SM1 returns, SM3 continues towards the door.
2. “Do you need some milk?”* a SM (SM4) from outside the dining room asks SM1. “Milk? (short pause in talk). No, thank you” she replies, continuing to look down at the trolley whilst talking.
3. Meanwhile, SM3 moves between be-served table and the buffet/area, but mostly keeps close to the be-served table. She has placed a cup and an orange on the opposite seat from where SM2 is seated.
4. Patient 1 is standing in front of the buffet. “There’s no more cultured milk”, she says. SM1 turns her head towards the door. SM4 (?*) SM4 (?). Can you fix some cultured milk?” Turns her body away from the buffet, quickly re-directs her gaze towards the buffet when done talking.
5. “Here you go”. SM 4 says.
6. Both SM1 and SM3 turns towards the SM 4, who is still out of view. SM3 grabs the carton and hurries down to a shelf and fetches a glass. SM 1 resumes overlooking the serving situation.
7. When the patients have finished at the trolley, SM 1 turns towards the self-serve table, where patients are getting seated.
8. SM 3 walks towards the be-served table. “Do you want something? [To eat*]”. SM2 asks SM3. “Go ahead. I just had lunch.” SM3 sits down.

The excerpt above exemplifies the start of the meal. The dining room appears quite busy. Staff members are moving in and out, and around the room (rows 1, 4, 5, 6). Under supervision from SM1, patients help themselves to food from the trolley or obtain their pre-prepared meals in the buffet/serving area (row 1). In the dining area, staff members position themselves around the table. SM2 sits down from the very beginning of the meal, and remains there until all patients are seated, and SM3 has already “found her seat” (rows 1, 7). SM3 is the last person to actually sit down at the table, but she “reserved” her seat when the meal began by placing her cup and an orange on the table (row 3). SM4, who is not assigned to participate in the actual meal, helps out by bringing missing items from the kitchen (row 4). Staff members’ verbal interaction is limited to short messages about practical issues (rows 2, 4, 6). Two of the staff members eat with the patients; the third grabs a fruit and hot drink (row 6). The overall structure of ‘pre-eating’ starts each meal, and the period lasts between 5 to 7 minutes. The closing of the dining room door indicates the end of pre-eating. Only those participating in the meal remain in the room. There are two leading structuring activities typical for ‘pre-eating’: *serving* and *positioning*.

### Serving

By deliberately positioning themselves close to the trolley and buffet, staff members can observe and supervise the patients. They can monitor activities, ensure that the patients take or get the correct amount and types of food, and safeguard according to each individual meal plans. The two serving stations, the trolley and buffet, are in close proximity, making it possible for one staff member to observe all patients simultaneously. During serving staff not participating in the actual meal contributes by obtaining missing food items from the kitchen, and sometimes solving ambiguity regarding the content or amount of a patient’s meal.

### Positioning

One staff member remains close to the activities in the serving area, and one staff member remains positioned near the dining table. By covering both areas, patients are always seen, enabling activities to happen simultaneously and patients to move between the two areas. This is exemplified in excerpt 1 during the interaction where SM3 stops in front of the trolley simultaneously as SM1 moves away from the trolley (row1). The staff member(s) close to the dining table observe and assist patients if necessary, and can intervene in cases of undesirable behaviour. Staff members occasionally swap positions. This exchange is rarely verbally communicated between staff members. The verbal communication between staff members during ‘pre-eating’ is quite limited, usually only short messages. In addition to the specific activities close to the serving area, a seating pattern in which staff reserve or seek the middle seats recurs during most of the observed meals. Positioning by “reservation” of seats using cups or food items is typical. Breaks in this pattern may happen if there is a change in the patient group or one staff member enters the room a bit late. The positioning becomes less ”active” and the regular reservation of seats occurs less frequently.

### Eating

The ‘eating’ period starts when all patients are seated. During this part of the meal, the activities are concentrated around the two tables in the dining area of the room (Picture 2). The transition between pre-eating and eating is not clear-cut. Patients start eating when they have served themselves or have obtained their pre-prepared meals, and do not wait for others or for staff members to be seated. The excerpt below is from a lunch meal. The meal has lasted for about 20 minutes. Two staff members, one nursing student, and four patients are seated at the “be-served” table. In this excerpt, the collaboration between staff members relates to management of a specific patient. Patient1 sits by the window, next to SM1, and SM1 is seated next to SM2. The three other patients are seated next to each other at the other side of the table. The nursing student is seated at the end of the table*.* Patient1 is quiet.

**Excerpt 2, illustrating*eating.***

Time span: 23.51-26.42.

1. SM2 initiates conversation. *“I overslept today”*, she says.*” Did you? But you weren’t late for work.* SM1 comments.
2. *“No, I have started to use my bike to work”* SM2 replies.
3. SMs and patients are engaged in conversation. Patient1 is quiet.
4. Patient1 starts to gather her things, and looks like she is preparing to leave the table. *“I have to go”*, she says. Both SMs and the student look in the patient’s direction.
5. *“Have you finished your glass of milk “*(?) SM1 asks.
6. *“Yes”,* the patient replies. There is still some milk left in the glass.
7. SM1 leans towards the patient. *“Make sure that you drink what’s left of it”* she says, in a matter-of fact- tone of voice. The everyday conversation between SMs and patients stops during this interaction.
8. Patient 1 finishes her glass of milk. *“Have a nice day”*, SM1 says. Patient 1 gets up.
9. *“Oh, that is right. You are going to a barista course. Will you make us some coffee when you get back (?)”* SM1 says, laughing.
10. * “Yes, I was thinking the same thing”* the nurse student says.
11. SM 2 at the end of the table follows the patient with her eyes. The patient moves to the door and leaves the room. The others continue their conversation.
12. SM 2 leans towards SM1. Talks to her in a low voice. *“Did she take her yogurt with her (?)*
13. * “Nope”* SM1 replies.
14. SM1 gets up and leave the room. She gets back in the room together with patient 1, who grabs her yogurt from the buffet, and they leave the room.
15. Staff and patient resume their “every day” conversation. SM1 returns a few seconds later, and joins the conversation.

The excerpt above exemplifies the main part of a meal. Staff members and patients are engaged in regular small talk (row 1). The seeming normality continues with conversation about SM2s morning. The conversation is interrupted when Patient1 prepares to leave, before the official ending of the meal (row 4). When the patient signals that she is about to leave, the dialogue between staff members and patients stops, and all attention is directed to patient1 (row 4). They remain silent until the situation is resolved (row 15). It takes a few moments before staff members remember why the patient is leaving: “Oh, that’s right” (row 9). SM2 chooses to address her colleague seated next to her and not the patient about the yogurt. She waits until the patient almost has left the room before she does so (row 12).

On average, the ‘eating’ event lasts between 18 – 24 minutes. The two main structuring activities are *division of labour* and *dialogue*.

### Division of labour

In most observed meals staff members sit facing each other, in contrast to the situation illustrated above, where staff members are seated next to each other. The seating arrangement affects the division of labour pattern between staff members. When staff members are seated facing each other, they more easily relate to each other and all patients. If something specifically happens during ‘eating’, it is always the staff member seated next to the patient that “handles” the situation, unless she chooses to involve her colleague by actively addressing her. When there is a break in this pattern, the division of labour is less obvious, and more communication between staff members is necessary to clarify who assumes responsibility for the patient, as illustrated in excerpt 2.

### Dialogue

The topics of conversation during mealtime are ‘small talk’ or dialogue concerning everyday matters, and dialogue directed specifically toward eating behaviour. This “two-layered” pattern was characteristic for most meals, as exemplified in excerpt 2. During the dialogue staff members switches from small talk to more “food oriented” talk, starting in row 5 with SM1s comment about the milk. Normal dialogue is initiated by either staff or patients in the beginning of “eating”. By keeping the topics neutral and impersonal, everyone can participate in conversation, if desired. When addressing eating-related matters, staff restricts conversation to the patient directly, in a lowered voice and often leaning towards the patient. The lower tone of voice, signals that the others are not “invited in”. By using humour to comment upon patient’s behaviour, like the nursing student in the excerpt above; the atmosphere in the dining room relaxes and seems less tense. Some patients choose to remain quiet throughout the meal or parts of the meal, such as Patient1 in the above example. Staff members refrain from commenting upon patients who remain quiet. They either continue speaking with other patients, or with each other if all patients are quiet.

### Completion

This last part of the meal, ‘completion’ , is relatively brief. The externally imposed structure informs the official ending, precisely 30 minutes after the official beginning of the meal. If all patients finish on time, staff and patients get up from the table and leave the room together. If the meal is delayed for some reason, it still ends 30 minutes after it started. In the excerpt below, the lunch is about to end. There are two staff members and four patients at the “be-served” table.

**Excerpt 3, illustrating*completion.***

Time span: 29:35–30:00.

1. All patients and both SM are engaged in dialogue about everyday matters.
2. When there is 30 seconds left of the meal, SM1 glances at the clock on the wall. *” Ok. Time is up. “* she says.
3. Three patients and SM1 instantly gets up from the table and leave the room.
4. All patients but one have finished their meals. Patient1 remains seated.
5. SM 2, seated next to patient1, gets up from the table, but sits down again when she sees that patient1 hasn’t finished her meal.
6. Patient1 leaves the table, and return with her nutritional drink – to replace what is left of the meal.

In this situation dialogue is interrupted by SM1’s comment about time being up, referring to the externally imposed time limit (row1). Patient1 has not yet finished her meal. When the rest of the group prepares to leave the room, both the patient and SM2 remain in the room (row2). There is no verbal communication between the SMs regarding who is to stay with Patient1. The dialogue continues almost until they leave the table.

The ‘completion’ event described above was characteristic for most of the observed meals. In addition, this is a quite short part, on average only lasting about 1 minute. The main activity is *end of meal preparations*.

### End of meal preparations

The completion of the meal is rarely stated verbally, except for short messages like “time’s up”. There are no closing remarks, like “thanks for the meal”. As the official ending of the meal approaches, staff members check the clock on the wall, but rarely their own watches. It is the clock on the wall that shows the “correct” or all agreed on time that informs the ending of the meal. Who is to stay behind with patients that have not finished eating is rarely stated or discussed. According to our observations this is most often the staff member seated next to the patient. Sometimes, the staff member that remains in the room and the staff member that leaves the room have a verbal exchange regarding practical issues.

**Figure 3:**
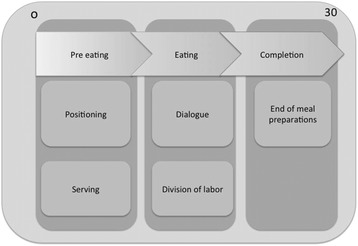
**The three events and main activities observed across meals.**

To summarize the results, the analysis of the observed meals reveals a clear internal structure. As presented above the observed meals were divided into three main events. The three events and accompanying main activities are summarized in Figure 3.

Activities reoccurred in a similar order across the data material, with specific characteristics within each event. These activities structured how the meals were managed by staff members participating in the meal.

## Discussion

Our results illustrate how the pattern of mealtime activities was structured into the three distinct main events: ‘pre-eating’ , ‘eating’ and ‘completion’. There were regular activities that occurred during each event, which provided a clear structure and routine associated with predictable staff behaviour and management of meals. The interactions between staff members maintain regularity during the meals. Across the data set, the activities revealed a clear internal meal structure, suggesting that staff members operated according to underlying rules, and that their responses were habituated. One way to understand habituated behaviour is to view these events as scripted behaviour and approach them from a script theoretical perspective [14,16,22]. The internal structure seen in sequential order and shared collective activity can be further understood if viewing the observed mealtime behaviour as actions in accordance with strong scripts [16]. The collective nature of the meal management, where several staff members participated and collaborated to perform the various activities, suggests interactive scripts. Interactive scripts are enacted when behavioural scripts are collectively engaged by two or more individuals [14,16]. During many of the observed activities, little verbal interaction between staff members seemed necessary for the activities to take place as expected. They had a shared understanding of the involved tasks to be performed within a “meal on EDU” script. Mangham [14] describes that two or more individuals involved in an activity for which interaction is required to accomplish a task, can create “interlocked behaviours” that are reciprocal and contingent on each other. Such accommodating processes can be viewed as concluding with the creation of a situational, appropriate pattern of events [11]. In the presented analysis, this interlocked behaviour was particularly evident during ‘pre-eating’. Staff members performed activities in different areas of the room, depending upon the other participants for all activities to be completed.

Meals are culturally recognizable events in social interaction [20]. The observed meals on the in patient EDU resembles the structure of a “normal” meal to a certain degree, in many ways mirroring how family meals are organized and carried out in western society. The meals are held in a dining room and people are eating together at the dining table(s). However, during the observed meals, staff members organized and managed the meals to a) facilitate meal completion and b) to achieve normalized eating behaviour. These two scripts occurred across all three events. The duality between the scripts was particularly evident during “eating”. Our findings suggest that the dialogue during the “eating” part of the meal also was two-layered. Staff members are switching between small talk, mirroring “normal” eating behaviour, and talk relating to the re-feeding goals underlying the meal, i.e. facilitating for meal completion. The observations illustrate how the two scripts occurred simultaneously and could be seen in their dialogue. Normal conversation dominated during “eating”. However, therapeutic dialogue placed normal conversation on hold and took precedence as soon as there was an incident challenging the goals, like the meal completion expressed by the re-feeding script. A possible explanation for such observations could be that staff members differed in how they balanced the script they used during “eating”. An enhanced awareness amongst staff members concerning which scripts are in play, and whether they are in agreement about the main tasks during a meal should be further explored. Staff members showed some differences in relation to the use of small talk during “eating”.

Some staff members continued talking if patients kept quiet, whilst others tuned down their own verbal activity when there was a lack of response. Some may have tried to mirror a normal meal by maintaining a conversation around the table, whilst other staff members might have used small talk as a distraction strategy to divert patients from their difficult feelings. Staff members have previously described different experiences with dialogue during the meal in terms of efficacy as a distraction strategy, and have used it inconsistently [9]. Patients have also expressed mixed views on the use of distraction during mealtimes. Sometimes it is experienced as helpful, and other times it can actually be counterproductive and hinder the eating process [8]. There is a need for further exploration of how dialogue is used therapeutically during mealtimes.

During the observed meals, there were hardly any discussions or disagreements directly between staff members and patients. Therefore, they showed little resemblance of being a battleground or power struggle as previously described in the literature [9,12,23]. Although the participating staff members and patients may experience the same situation differently, a strongly scripted internal structure can help facilitate meal management in a manner that minimizes the need for discussions and disagreements between staff and patients. Nurses underline the importance of clear eating rules when eating with ED patients [13]. Such structuring arrangements can reduce the feeling of power struggle or battleground since the scripts gives direction for the acceptable behaviour and activities.

Participating in, and managing a meal has been described as difficult by staff members [9,23], and the use of strong scripts might ease this experience. A clear understanding of how the meal should proceed among staff members and patients, is likely to lead to fewer discussions and less negative feelings around meals for both staff members and patients. A mutual understanding about the internal structure and expected activities might also ease teamwork and collaboration during meals, exemplified in how staff divided labour during eating. This is a theme that has been emphasized as essential for a smooth running of meals [9]. However, strongly scripted behaviours have been accused of being mindless, in the sense that they are performed automatically [24]. A possible challenge can therefore be if behaviour becomes excessively scripted, or if the importance of fulfilling scripts leaves little room for flexibility in meal management or the individualized treatment of each patient. Seeing the individual is considered important by patients on inpatient EDUs [25]. Little attention to the individual perspective combined with a ritualized meal structure can add to felt detachment and distance during mealtimes [8]. However, Gioa and Poole [16] point out that such behaviour is not necessarily unconscious, hence, all behaviour during particular events are not scripted. Scripts can also serve to conserve cognitive capacity. Consequently, there is more space to handle events not included in the scripts [24], allowing staff members to perform several tasks simultaneously during mealtimes, like focus on patients’ needs, participate in dialogue and simultaneously observe each patient’s eating behaviour.

### Strengths and weaknesses of the study

The opportunity to conduct an observational study like the present study, and videotape meals to study SMs teamwork and collaboration has some obvious strengths and weaknesses. First, the first and second author of this paper, have long-standing clinical experience treating ED patients. The first author has several years of experience participating in meals at this EDU. This was an advantage by easing or normalizing the interaction between staff members and patients during data collection. Awareness of the video recording of the mealtimes could potentially influence staff members and patients’ behaviour during the meal situation. To minimize intrusiveness for staff members and patients, researchers were not present during the recordings. This strategy helped reduce potential bias from influencing participants’ behaviour by awareness of additional persons present or being filmed [20].

Since we were interested in the staff interactions for meal management, we chose to record the table where they were seated. In most cases that was the “be served” table. This may have had some impact on the findings, as patients seated at the “self-serve” table tend to need less support from staff member when seated, which may affect how staff manages the meal. However, the internal structure of the meal, and the staffs’ activities is to a large degree similar for the two tables. During the period of data collection, we anticipate that staff and patients became habituated to the video camera, since there was no operator behind the camera [26]. The density and permanence of video data enhanced the credibility of the study by making it possible to review the observed situations numerous times, observe more and re-review all aspects of the situations [19,26]. Although qualitative methods potentially do not form a representative view of the topic, qualitative data is acknowledged as valuable in enhancing the understanding of the phenomenon being studied.

## Conclusions

In this study, we found that mealtime management on the observed inpatient unit had a clear internal structure, comprising three main structuring periods: pre-eating, eating and completion. We found that across meals, this main structure remained, and that the staff behaviour reflected two mutually constitution scripts.

Staff management of mealtimes on EDUs is an interactional activity. Increased staff focus on how meals are managed and which scripts are in play, may help staff members to further explore their behaviour and collaboration during mealtimes, and also contribute to improved awareness in interaction with patients during the various phases of the meal. Enhanced awareness around potentially competing, operating scripts is important when developing new approaches or a possible change in mealtime practice on EDUs. Also, knowledge about how meals on EDUs are managed and carried out is important in order to develop future interventions to support patients during mealtimes. Additionally, more knowledge amongst clinicians concerning the internal structure of a meal is important in order to assess and evaluate own practice.

## Abbreviations

AN: Anorexia nervosa
ED: Eating disorders
EDU: Eating disorder unit
SM: Staff member
